# Determination and analysis of the complete mitochondrial genome of *Barilius barila* (Cypriniformes: Danionidae: Chedrinae)

**DOI:** 10.1080/23802359.2022.2148824

**Published:** 2022-11-21

**Authors:** Xiao Jiang Chen, Lin Song, Yang Song, Quan Wang

**Affiliations:** College of Fisheries Science and Technology, Jiangsu Agri-animal Husbandry Vocational College, Taizhou, P.R. China

**Keywords:** Mitochondrial genome, *Barilius barila*, Chedrinae, *Opsarius canarensis*, *Opsarius caudiocellatus*

## Abstract

Cyprinid fish *Barilius barila* found in the Irrawaddy water system is a valuable fishery resource and has been listed as Least Concern by the IUCN. This study determined the complete mitochondrial genome of *B. barila* from Yunnan, China, for the first time. Circular molecule of *B. barila* mitogenome was sequenced to be 16,560 bp in length, with the typical gene structure of 13 protein-coding genes, 22 transfer RNA genes, two ribosomal RNA genes, and two noncoding areas (control region and the origin of L-strand replication). Overall nucleotides composition appeared to be 27.5% A, 24.8% T, 19.2% G, and 28.6% C, with a slight AT (52.3%) bias. The topology of the phylogenetic tree showed that *B. barila* was well grouped with *Opsarius caudiocellatus,* and clustered together with the genus *Opsarius* instead of *Barilius,* revealing that it was more reasonable for *Barilius barila* to belong to *Opsarius* rather than *Barilius*.

## 1. Introduction

**Figure 1.**
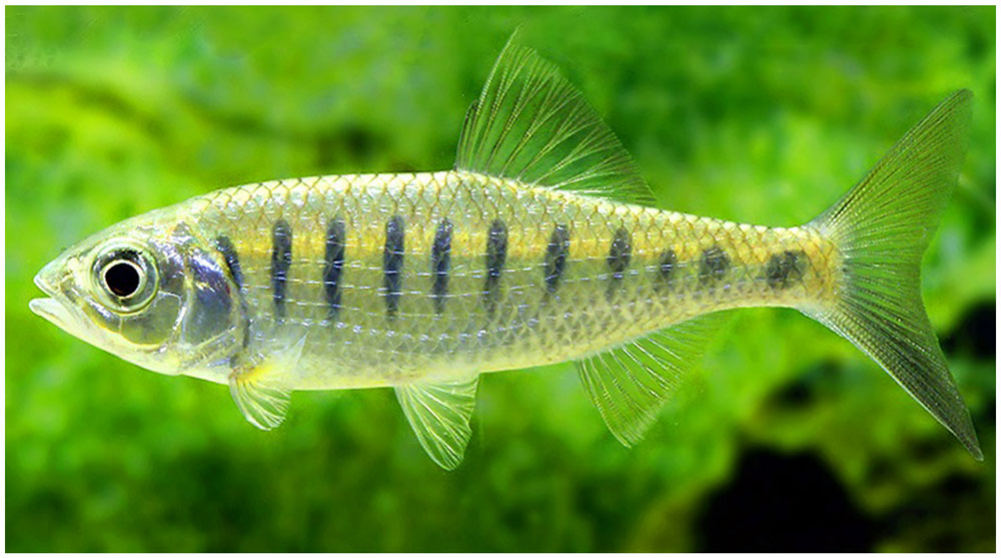
*Barilius barila*, the specimen was collected from the Da Ying River in Yingjiang County, Yunnan Province of China. Photographs by Xiao Jiang CHEN on 15 June 2021.

The cyprinid fish, *Barilius barila* (Hamilton, 1822) belongs to the Chedrinae subfamily of the Danionidae family and is endemic to the Irrawaddy water system with ornamental and economic value. It inhabits large hill streams and shallow clear rivers along foothills. It can be easily distinguished based on the morphological features as follows (Figure 1): dorsal fin iii-7-8, anal fin ii-10-11, pectoral fin i-11-12, ventral fin i-7-8; 22 predorsal scales, 40–42 + 2–3 lateral line scales, pectoral fin as long as head, silvery white body with 11–15 blue patches on both sides (Chu 1984; Prabhu et al. 2020). Due to a great reduction in natural population number, *B. barila* has been assessed as Least Concern (LC) status in the IUCN Red List of Threatened Species in 2010. Recently, there were few *Barilius* species mitogenome sequences reported in public. The present study examined the complete mitochondrial sequence of *B. barila* and investigated the phylogenetic relationships within Chedrinae for the first time, which would be advantageous in DNA barcode development and targeted conservation.

## 2. Materials and methods

### 2.1. Sample collection and preservation

Experiments in this study complied with the recommendations of the Ethics Committee for Animal Experiments of Jiangsu Agri-animal Husbandry Vocational College. The *B. barila* sample was obtained from Da Ying River in Yingjiang County, Dehong Prefecture, Yunnan Province of China (24°69′05.95″ N, 97°94′89.69″E) on 8 July 2022. The obtained fish specimens were euthanized, first anesthetized using a concentration of 0.2 mL/L eugenol solution, then placed into 75% ethanol for fixation and finally transferred to 95% ethanol for long-term storage. All specimens were deposited in the fish collection at Aquatic Science and Technology Institution Herbarium (https://www.jsahvc.edu.cn/; Deposit number ASTIH-21b1108d28; Chen Xiao Jiang, 2007020030@jsahvc.edu.cn).

### 2.2. Mitochondrial genome sequencing and phylogenetic analysis

The muscle tissue was collected for genomic DNA isolation using the Tguide Cell/tissue genomic DNA Extraction Kit (OSR-M401) (Tiangen, Beijing, China). After DNA sample quality control, a DNA library was constructed and amplified by PCR, followed by size selection and library quality check, finally the amplified original library DNA was subjected to Illumina HiSeq 4000 Sequencing platform (Illumina, CA). The sequenced fragments were processed for the quality check to filtrate low-quality reads on FastQC Version 0.11.8 (Andrews 2015), and then assembled into a circular mitogenome of *B. barila* by MetaSPAdes 3.13.0 (Nurk et al. 2017) with *Barilius malabaricus* MN650735 as reference, and then the assembled mitochondrial genome sequences were annotated using MitoMaker 1.14 (Bernt et al. 2013) with default parameters. The resulting complete mitochondrial genome was deposited in GenBank with accession number OM617728. OGDRAW (https://chlorobox.mpimp-golm.mpg.de/OGDraw.html) was used to draw the genome maps (Greiner et al. 2019). Alignments, analysis, model calculation, and phylogenetic reconstruction were all completed by MEGA X (Kumar et al. 2018).

## 3. Results and discussion

### 3.1. Mitochondrial DNA genome structure

**Figure 2.**
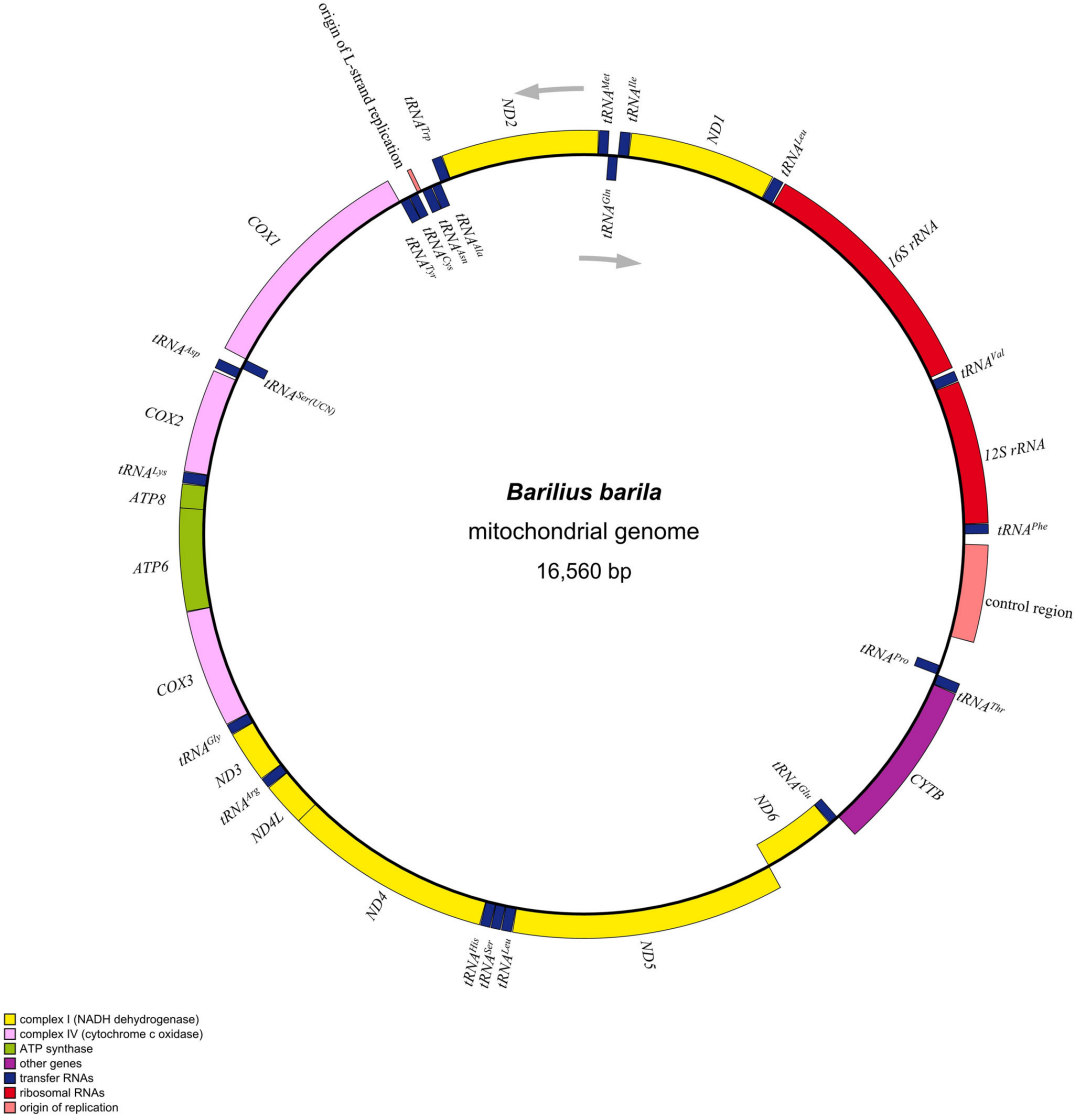
Mitochondrial genome map of *Barilius barila* (Genbank no. OM617728), consisting of 13 PCGs, 22 tRNAs, two rRNAs, an origin of L-strand replication and a control region. The arrows represents direction of transcription. *tRNA^Gln^*, *tRNA^Ala^*, *tRNA^Asn^*, *tRNA^Cys^*, *tRNA^Tyr^*, *tRNA^Ser(UCN)^*, *tRNA^Glu^*, *tRNA^Pro^*, and *ND6* encoded on L-strand are displayed inside the circle, the other genes encoded on H-strand are displayed outside the circle.

The *B. barila* mitogenome had a closed double-stranded circular molecule with 16,560 bp in length and essentially contained 13 protein-coding genes, 22 transfer RNA genes, two ribosomal RNA genes, and two noncoding areas (a control region and an origin of L-strand replication), resembling those of other Danionidae species (Chen et al. 2022; Song et al. 2022). The overall nucleotide contents of A, T, G, and C appeared to be 27.5%, 24.8%, 19.2%, and 28.6% respectively, thereby with a slight AT (52.3%) bias. Most mitochondrial genes were encoded on H-strand except for *ND6* and eight tRNA genes (*tRNA^Gln^*, *tRNA^Ala^*, *tRNA^Asn^*, *tRNA^Cys^*, *tRNA^Tyr^*, *tRNA^Ser(UCN)^*, *tRNA^Glu^*, and *tRNA^Pro^*), which were encoded on the L- strand (Figure 2). All PCGs initiated with normal ATG except for *CO1* with GTG as its start codon. Besides, the stop codon usage patterns were diverse: most PCGs (*ND1*, *CO1*, *ATP8*, *ATP6*, *CO3*, *ND3*, *ND4L*, and *ND5*) terminated with routine TAA codon, and two genes (*ND2* and *ND6*) utilized TAG as the stop codon, while the remaining three PCGs ended by incomplete TA (*ND4*) or single T (*CO2* and *Cytb*), which was a common feature among vertebrate mitogenomes (Luo et al. 2019; Tan et al. 2020). Sixteen intergenic spacers were found in the whole mitogenome, ranging from 1 to 33 bp in length. Simultaneously, ten reading frame overlaps were observed with the largest overlap of 7 nucleotides in two sites (*ATP6-ATP8* and *ND4L-ND4*). The size of 22 tRNA genes varied from 66 bp (*tRNA^Cys^*) to 75 bp (*tRNA^Lys^*), while the control region extended up to 382 nucleotides and was identified between *tRNA^Phe^* and *tRNA^Pro^*. The subunits of rRNA in *B. barila* genome were of two types, namely *12S rRNA* and *16S rRNA*, with lengths of 954 bp and 1653 bp respectively.

### 3.2. Phylogenetic analysis

**Figure 3.**
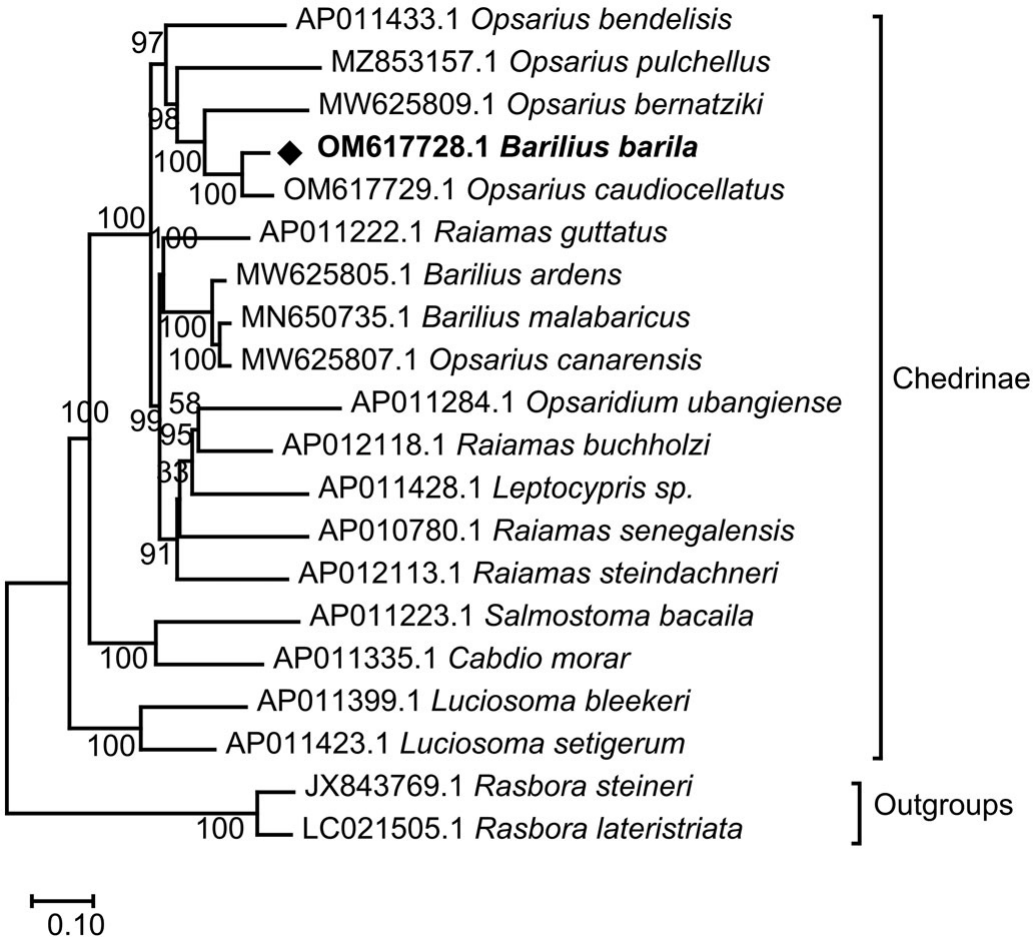
Maximum-likelihood (ML) phylogenetic tree reconstructed using concatenated mitochondrial protein-coding genes of *B. barila* and other 19 fish species. Accession numbers were followed by the name of the species. The tree topology was evaluated by 1000 bootstrap replicates. Bootstrap values at the nodes correspond to the support values for ML methods.

Amino acid sequences of 13 protein-coding genes of *B. barila* were aligned on MEGA X with that of other 19 species of fish from 9 genera (*Opsarius, Barilius, Raiamas, Opsaridium, Leptocypris, Salmostoma, Cabdio, Luciosoma, Rasbora*) available in Genbank (Tang et al. 2010; Saitoh et al. 2011; Chang et al. 2013; Kusuma and Kumazawa 2016; Miya et al. 2016; Prabhu et al. 2020; Chen et al. 2022; Yu et al. 2022). *Rasbora lateristriata* and *Rasbora steineri* were selected as outgroups. The best evolutionary model was simulated to be GTR + G + I for it has obtained the lowest Bayesian information standard scores (Nei and Kumar 2000). The ML analysis generated topological structure and the phylogenetic position of *B. barila* in subfamily Chedrinae was shown in Figure 3. All 18 Chedrinae fish species were split into three well-supported major clades, Clade A (A1 + A2 + A3) and Clade B and Clade C. Clade A2 (*Raiamas guttatus, Barilius ardens, Barilius malabaricus,* and *Opsarius canarensis*) became the sister-group with the Clade A3 (*Opsaridium ubangiense, Raiamas buchholzi, Leptocypris sp, Raiamas senegalensis,* and *Raiamas steindachneri*), and they formed a sister-group with the Clade A1 (*Opsarius bendelisis, Opsarius pulchellus, Opsarius bernatziki, Barilius barila, and Opsarius caudiocellatus*). Further, it showed that *B. barila* was clustered together with genus *Opsarius,* especially well grouped with *Opsarius caudiocellatus.*

The classification of the genus *Barilius* was very complex. *Barilius* (sensu lato) was divided into the genera *Barilius* and *Opsarius* by Howes (1980). Qin et al. have proposed that the genus *Barilius* from South Asia should be changed to Opsarius based on morphology and molecular phylogeny, such as *Barilius canarensis* was changed to *Opsarius canarensis* (Qin et al. 2019). The phylogenetic tree showed that it was more reasonable for *Barilius barila* to belong to the genus *Opsarius*. In addition, *Opsarius canarensis* was clustered together with the genus *Barilius* instead of *Opsarius,* which was incongruent with the viewpoint of Qin et al. It was recommended that more data be needed for further analysis and confirmation.

## 4. Conclusions

This study determined the complete mitochondrial genome of *B. barila* for the first time using the high-throughput sequencing technology. The assembly circular mitogenome was 16,560 bp long (deposited in GenBank with accession number OM617728). The phylogenetic tree was constructed based on the maximum likelihood method showed that *B. barila* was clustered together with the genus *Opsarius*, revealing that it may be more reasonable for *B. barila* to belong to the genus *Opsarius* instead of *Barilius.* This mitochondrial genome would establish a basis for furthering research on species evolution and phylogenetic of the subfamily Chedrinae, it would also be conducive to the development of the DNA barcode and targeted conservation.

## Ethical approval

Experiments were performed in accordance with the recommendations of the Ethics Committee for Animal Experiments of Jiangsu Agri-animal Husbandry Vocational College. These policies were enacted according to the Chinese Association for the Laboratory Animal Sciences and the Institutional Animal Care and Use Committee (IACUC) protocols.

## Supplementary Material

Supplemental Material

## Data Availability

The genome sequence data that support the findings of this study are openly available in GenBank of NCBI at (https://www.ncbi.nlm.nih.gov/) under the reference number OM617728. The associated “BioProject”, “Bio-Sample” and “SRA” numbers are PRJNA808197, SAMN26036042, and SRR18066595 respectively.
