# Supplementary figures and images for: Determination and analysis of the complete mitochondrial genome of Barilius barila (Cypriniformes: Danionidae: Chedrinae)
Source: Mitochondrial DNA B Resour. 2022 Nov 21;7(11):2002–5. doi: 10.1080/23802359.2022.2148824 (PMC9704074; doi:10.1080/23802359.2022.2148824)

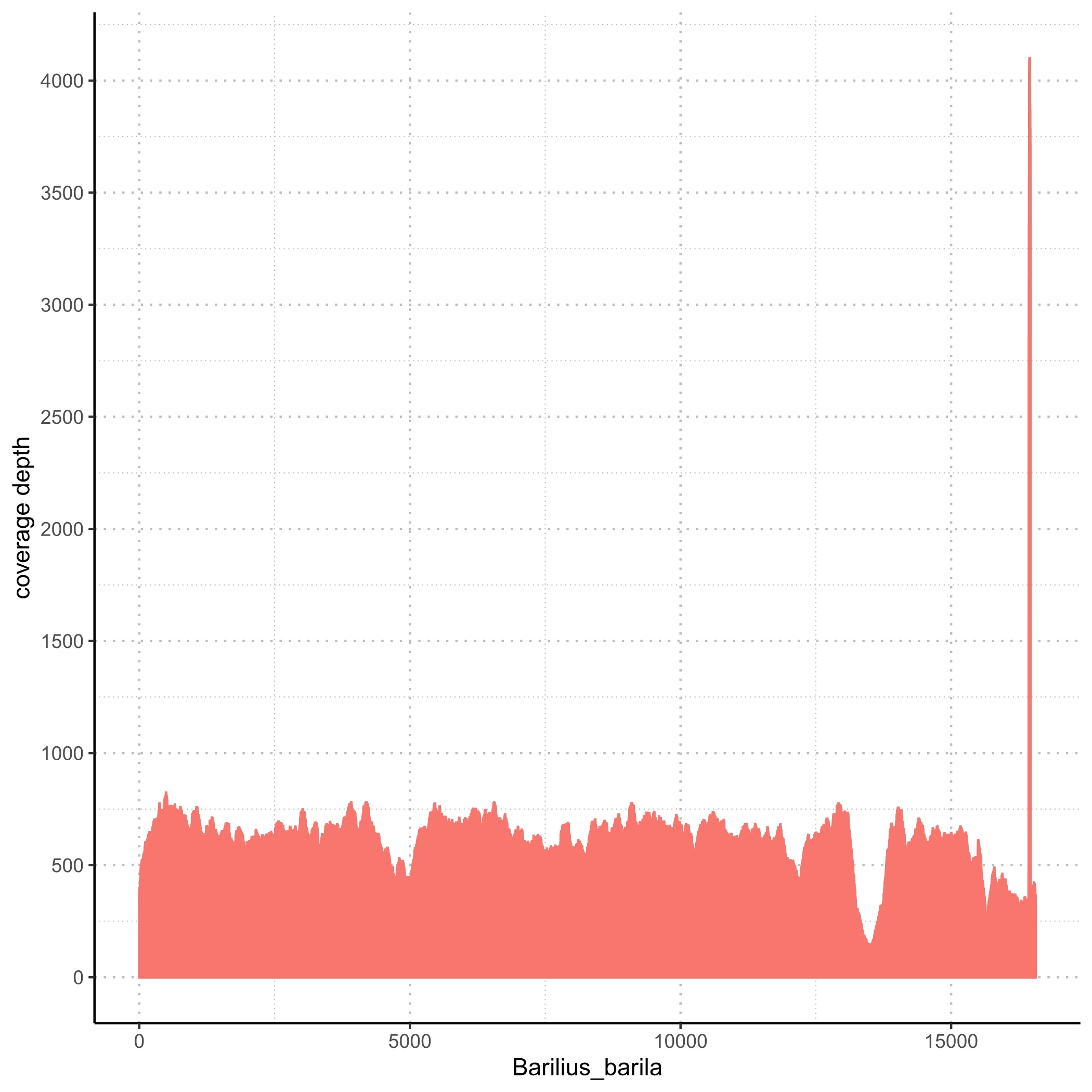

Supplement: Supplemental Material [file TMDN_A_2148824_SM2919.jpg]
